# Micro-Computed Tomography Evaluation of Minimally Invasive Shaping Systems in Mandibular First Molars

**DOI:** 10.3390/jcm11154607

**Published:** 2022-08-08

**Authors:** Elio Berutti, Edoardo Moccia, Stefano Lavino, Stefania Multari, Giorgia Carpegna, Nicola Scotti, Damiano Pasqualini, Mario Alovisi

**Affiliations:** Department of Surgical Sciences, Dental School, University of Turin, 10125 Turin, Italy

**Keywords:** microtomography, molar, root canal therapy, dental pulp cavity, mechanical preparation, rotary instruments

## Abstract

The aim of this study was to compare the shaping ability of a modified ProTaper Next technique (PTNm) with that of TruNatomy (TN) in lower molars mesial curved canals using micro-computed tomography (Micro-CT). Sixty mesial canals of first mandibular molars were randomly assigned between two groups (n = 30). After canal scouting with K-File #10, glide path and shaping were carried out with TN or PTNm systems. The PTNm sequence consists of ProGlider, followed by ProTaper Next X1 and apical finishing with NiTiFlex #25 up to working length (WL) to ensure adequate apical cleaning. Samples were scanned using micro-CT and pre- and post-shaping volumes were matched to analyse geometric parameters: the volume of removed dentin; the difference of canal surface; centroid shift, minimum and maximum root canal diameters; cross-sectional areas; the ratio of diameter ratios (RDR) and the ratio of cross-sectional areas (RA). Measurements were assessed 2 mm from the apex and in relation to the middle and coronal root canal thirds. Data were analysed using ANOVA (*p* < 0.05). No statistically significant differences were found between the groups for any parameter at each level of analysis, except for RA at the coronal level (*p* = 0.037). The PTNm system showed the tendency to enlarge more in the coronal portion with a lower centroid shift at apical level compared with TN sequence (*p* > 0.05). Both PTNm and TN sequences demonstrated similar maintenance of original anatomy during the shaping of lower molar mesial curved canals.

## 1. Introduction

The primary purpose of endodontic treatment is decontamination of the root canal system from bacteria, pulp residues, organic substrates and shaping debris, while maintaining the root canal anatomy [1]. Establishing a hermetic seal via three-dimensional (3D) filling is mandatory to complete endodontic disinfection [1,2,3]. 

Root canal preparation is usually divided into different steps: canal scouting with stainless steel K-Files #08–10 provides the initial patency, while the subsequent glide path minimizes the risk of placing torsional stress on the shaping instruments [4,5]. Glide path and shaping procedures require the use of manual or mechanical nickel-titanium (NiTi) instruments [6], of which the latter may reduce operative time and canal transportation compared with manual instruments, regardless of operator experience [7].

Modern endodontic techniques aim to simplify clinical procedures while optimizing long-term tooth prognosis [8]. Therefore, endodontic instruments have been designed to display less taper, exhibit greater flexibility due to metallurgical properties, and shorter sequences with enhanced cyclic fatigue resistance compared with traditional instruments [9]. 

ProTaper Next (PTN) rotary shaping instruments have a M-wire alloy, a rectangular section and an asymmetrical rotation center which provides a ‘swaggering’ movement. These features reduce contact between the instrument and the canal walls, facilitate efficient debris removal and a give greater flexibility than previous generation shaping instruments [10,11,12,13]. TruNatomy (TN) describes a novel sequence of NiTi instruments with post-manufacturing thermal treatment, an off-centred parallelogram cross section, a regressive taper, and a small initial wire blank of 0.8 mm diameter in the shaping files [14,15]. 

The aim of this study was to evaluate a modified ProTaper Next sequence (PTNm) as a shaping technique for use in narrow canals and long and thin roots with accentuated curvatures. The modified sequence consists of ProGlider, followed by PTN X1 instrument and apical finishing with NiTiFlex #25 up to working length (WL) to ensure adequate apical cleaning. This technique was compared with TN shaping with Prime instrument, due to their ability to shape difficult anatomies and the micro-computed tomography (micro-CT) analysis of the resultant post-shaping geometries was performed.

The study was designed to test the null hypothesis that the TN technique and the PTNm sequence would not differ in their abilities to preserve the original root canal anatomy during the shaping of curved lower molar mesial canals.

## 2. Materials and Methods

### 2.1. Samples Selection

Mandibular first molars with a fully formed apex that had not previously undergone endodontic treatment were selected in accordance with the local ethics committee (Protocol number CS2_1053_2022). The teeth were extracted for periodontal reasons, and they were free of caries, cracks, and artificial alterations. A sample size of 30 per group was calculated with G*Power 3.1.4 (Kiel University, Kiel, Germany) considering alpha-error = 0.05 and ß = 0.95. 

After root debridement with Gracey curette 7/8 (Hu-Friedy, Chicago, IL, USA), the specimens were immersed in 0.01% NaOCl (Niclor 5, OGNA, Muggiò, Italy) at 4 °C for 24 h before storage in saline solution. The teeth were placed on a customized support to perform a preliminary low resolution micro-CT scan to obtain an overall outline of the canal anatomy and to select teeth that met the inclusion criteria (SkyScan 1172, Bruker micro-CT, Kontich, Belgium). Preliminary scans were conducted as follows: 450 projections through a 225° rotation (180° plus cone angle of the X-ray source) using a 1.0 mm thick aluminum filter, voltage = 100 kV, current = 80 μA, source-to-object distance = 80 mm, source-to-detector distance = 220 mm, pixel binning = 8 × 8, exposure time/projection = 0.2 s. Axial sections were reconstructed with isotropic voxels and morphological parameters of the mesial canals were obtained. Mesial-separated canals measuring 12 ± 2 mm from the canal orifice to the apical foramen, with 20°–40° primary mesio-distal curvature, 10°–30° bucco-lingual canal curvature and 4 < r ≤ 8 mm main curvature radius were selected [16,17]. The point of maximum curvature was located within the middle third of each root canal. Teeth with confluent canals, accentuated isthmuses or significant calcifications were excluded, as were any that did not concur with the above inclusion criteria. Of 48 teeth assessed for eligibility, 18 were excluded due to anatomical features, and 30 were included in the study, each with two separated mesial canals for a total of 60 mesial canals equally distributed in two groups. 

### 2.2. Samples Preparation

Both mesial canals in each sample were shaped with one of the tested techniques. The mesio-lingual (ML) and mesio-buccal (MB) canals were randomly assigned to shaping with PTNm (n = 30) or TN (n = 30) using a computer-generated randomization system. Instrumentation was carried out by a single expert operator skilled in both techniques and calibrated for pecking motion amplitude and pressure on the handpiece. As shaping sequences require specific settings and techniques, it was not possible to blind the operator. However, a single operator, blinded to the aim of the study, checked randomization, allocation, and performed the statistical analyses. 

The traditional access cavity was prepared, and canal scouting was accomplished in all mesial canals with #10 K-File at WL using Glyde (Dentsply Sirona, Ballaigues, Switzerland) lubricating gel (0.80 mg). WL was established with 10X magnification (OPMI Pro Ergo, Carl Zeiss, Oberkochen, Germany) when the tip was visible at the apical foramen. 

In the PTNm group, the glide path was performed with rotary single file ProGlider (PG) (0.16, taper from 0.02 to 0.085) (Dentsply Sirona Maillefer) and shaped with PTN X1 (0.17, taper from 0.04 to 0.075) (Dentsply Sirona Maillefer) up to WL. Both PG and PTN X1 utilized an endodontic X-Smart Plus engine (Dentsply Sirona, Ballaigues, Switzerland) with 16:1 contra angle (300 rpm, 4 Ncm) in continuous rotation up to WL. Apical finishing was then achieved manually with a NiTi-file #25 (Dentsply Sirona Maillefer) with a ‘feed-it and pull’ movement to WL.

In the TN group, pre-flaring was performed with TN Orifice Modifier (020, taper 0.08) (Dentsply Sirona Maillefer) and glide path was achieved with the TN Glider (017, taper 0.02) (Dentsply Sirona Maillefer). Root canal shaping was completed with TN Prime (026, taper 0.04 variable) up to WL. Each instrument utilized an endodontic X-Smart Plus engine (Dentsply Sirona, Ballaigues, Switzerland) with 16:1 contra angle (500 rpm, 1.5 Ncm) in continuous rotation up to WL, according to manufacturer’s instructions. 

New instruments were used for each canal (30 sets of instruments per group) and operated according to the manufacturer’s instructions using ‘in and out’ movements without intentional brushing effects. 

Irrigation was performed without engaging canal walls up to 4 mm from the WL using a manual syringe with a dedicated 30 G flexible endodontic needle, alternating 5% NaOCl and 10% EDTA, to a total of 10 mL per sample. Recapitulation with a #10 K-File was conducted between each instrument. The samples were then stored in saline solution prior to micro-CT scanning.

### 2.3. Micro-Computed Tomography Analysis

The samples underwent high resolution scanning before and after instrumentation to analyze geometrical modifications to the root canal (SkyScan 1172^®^: © Bruker microCT, Kontich, Belgium). Samples were mounted on a customized support and micro-CT scans were performed at 100 kV and 100 μA, at an isotropic resolution of 15 μm/pixel, over approximately 4 h and 2 min for each. The scans were performed with a rotation step of 0.2° and a frame averaging of 4, with a 360° full rotation, using an aluminium and copper filter for beam hardening. Each scan produced 3600 cross-sections per sample, at a resolution of 1000 × 666 pixels. The canal paths were analysed with high resolution 3D renderings through orthogonal axial sections to ensure the homogeneity of the samples at baseline.

The images were reconstructed using the NRecon software (SkyScan 1172, Bruker micro-CT, Kontich, Belgium) with standard parameters for beam hardening and ring artifact correction. The bi-dimensional (2D) and 3D root canal geometrical parameters were calculated using the Materialize Mimics 20.0 software (Materialize NV, Leuvren, Belgium), reducing manual bias. The increase in canal volume and surface area was calculated for each sample through 3D renderings. Bi-dimensional parameters were measured starting from orthogonal cross sections: root canal centroid shift, the ratio of diameter ratios (RDR), and the ratio of cross-sectional areas (RA)[9,18] RDR represents the tendency of an instrument to asymmetrically enlarge the root canal in one direction: RDR = (D/d)post/(D/d)pre, where (D/d)post is the post-preparation ratio of the major diameter (D) to the minor diameter (d) and (D/d)pre is the pre-preparation ratio of D to d. Therefore, when the values are close to 1, they represent greater maintenance of the original canal geometry. RA quantifies the ability of an instrument to enlarge the root canal space: RA = Apost/Apre, where Apost and Apre are the post-preparation and the pre-preparation cross-sectional areas, respectively. Values closer to 1 correspond to a smaller difference between pre- and post-instrumentation measurements, indicating in a more conservative instrumentation [7,19]. Root sections orthogonal to canal axis were set at three different levels of analysis: apical (A, 2 mm from the apical foramen), middle (M, set at the point of maximum curvature), and coronal (C, set in correspondence to the middle portion of the root canal coronal third defined by 3D calculation of the root canal length from apex to orifice). An automated minimum threshold was set to avoid manual errors [18,20].

### 2.4. Statistical Analyses

The normal distribution of the data was analyzed with a Shapiro–Wilk normality test. Geometrical differences at baseline between groups were analyzed with a Kruskal–Wallis and post hoc Dunn’s tests (level of significance: *p* < 0.05). One-way ANOVA and post hoc Turkey–Kramer tests were used to analyze the increase of canal surface area and volume, the centroid shift, and the impact of instrumentation on RDR and RA at each level of analysis (*p* < 0.05). All the statistical analyses were conducted with the Minitab 15 software package (Minitab Inc., State College, PA, USA).

## 3. Results 

**Figure 1:**
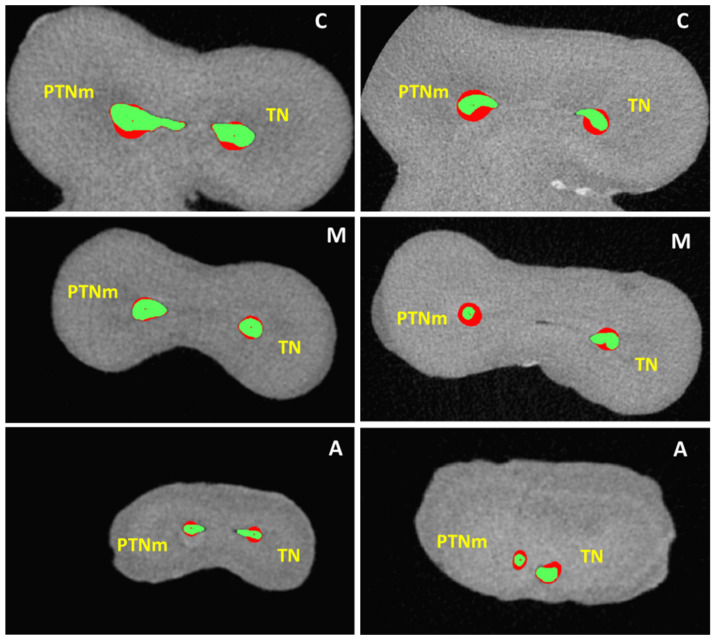
2D matching of pre-operative (green) and post-shaping (red) canal sections at the apical (**A**), point of maximum curvature (**M**), and coronal (**C**) levels of analysis in both groups.

**Figure 2:**
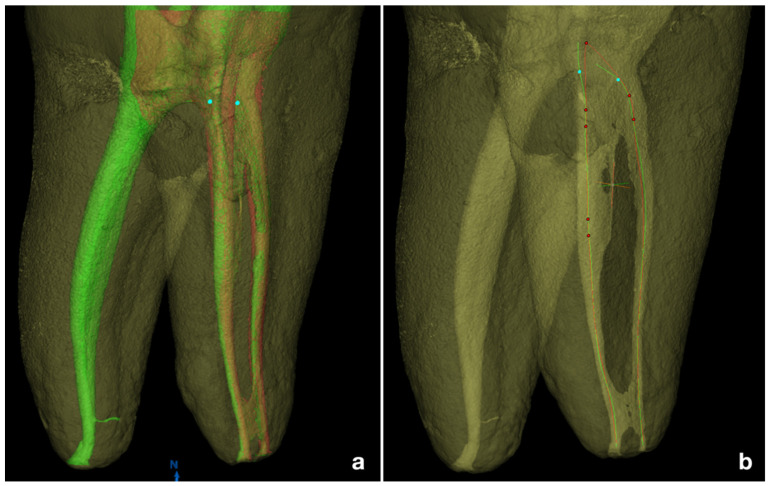
(**a**). 3D matching of pre-operative (green) and post-shaping (red) canal volumes, and (**b**). the pre-operative (green) and post-shaping (red) root canal centroids in the mesial canals.

The mean (±standard deviation) canal curvature was 32.1° ± 2.2° (min = 25°, max = 37°) in the PTNm group and 31.3° ± 1.9° (min = 24°, max = 35°) in the TN group, with no statistical difference between the groups (*p* = 0.12). Similarly, the radii of curvatures showed no differences between groups (*p* > 0.05). Baseline canal volume, surface area, and apical diameter also displayed homogeneity between groups (*p* > 0.05) and are presented in Table 1. The 3D and 2D pre- and post-operative geometrical parameters are shown in Table 2. No statistically significant differences were found between the groups for any parameter at each level of analysis, except for RA at the coronal level (*p* = 0.037). A higher tendency to remove dentin was observed in the PTNm group, especially in the coronal portion. The TN sequence demonstrated a higher centering ability at coronal level (*p* > 0.05), while PTNm system showed a lower centroid shift in the apical third (*p* > 0.05) (Figure 1 and Figure 2). No instruments were fractured during instrumentation.

## 4. Discussion

The purpose of this study was to evaluate the outcomes of root canal preparation of severely curved canals using two different shaping sequences with distinct taper and design but leading to similar apical size. Both tested shaping systems produced a well-centred preparation that respected the original canal anatomy, and the null hypothesis was generally accepted. 

The PTNm technique utilizes a manual #25 NiTiFlex file for the finishing of the apical third. The use of this flexible 0.02 taper finishing file is tried to maintain canal curvatures minimizing apical transportation. This preserves the original canal anatomy by reducing excessive canal instrumentation in complex cases with pronounced curvatures, while ensuring the effectiveness of irrigant cleansing [21,22,23]. Nevertheless, it has been demonstrated that an apical diameter #25–30 is necessary to rise the efficacy of the endodontic irrigants, without the need to increase the taper during shaping [21,22,23]. Therefore, the possibility of maintaining lower-tapered preparations is dependent on the maintenance of an adequate apical diameter, especially in highly curved canals. However, the rationale for the use a PTNm sequence could be related to the use of one single system for the shaping of curved mesial and oval distal lower molar canals without the risk not to touch the coronal root canal portions.

An extracted tooth model is usually transferable to the clinical setting and pre-operative homogeneity between samples is essential to ensure an adequate standardization [7,24]. In this micro-CT study, baseline homogeneity for 3D and 2D parameters was assumed, in agreement with previous observations [25]; however, the small sample number could be considered a limitation. Micro-CT analysis enables the non-invasive evaluation of pre- and post-operative root canal morphology, being an effective indicator of instrument shaping ability [7,26,27]. In this study, the superposition of the scanned volumes and the analysis of the root canal cross sections resulted in an accurate comparison of the shaping outcomes for the tested instruments in the apical, maximum curvature, and coronal levels of lower molar mesial canals [28]. These levels were selected as those most representative of the critical shaping portions [29], especially in the lower molar mesial canals that tend to require the most involved endodontic treatment of any teeth, with their complex anatomy often leading to procedural aberrations [30]. 

Root canal transportation can occur during endodontic treatment involving excessive dentin removal [31]. Furthermore, the straightening of the canal curvature leads to a reduction in the thickness of the dentinal walls and reduces the long-term prognosis of a tooth [1,29,30,31,32]. Therefore, the maintenance of the distal coronal third of the mandibular molar mesial root, known as the ’danger zone‘, is an important prognostic factor [33,34]. 

In this study, an intentional brushing motion was avoided, and a glide path was created to reduce the volume of dentin removed and to decrease the number of pecking motions required to reach the full WL [7,35,36]. Gel-chelating agents were used for canal scouting, while 10% EDTA and 5% NaOCl were used as alternating irrigants during glide path and shaping. This irrigation protocol replicated previously reported experimental conditions, even if the effects of different EDTA concentrations on shaping outcomes remain unclear and may represent a limitation of this study [12,37].

The two tested techniques are clinically indicated for the shaping of narrow and severely calcified canals, long and thin roots and accentuated curvatures, due to their advantage of imparting a low taper to canals. Generally, it is necessary to use carrier-based obturation techniques or single cone with bioceramic sealers to fill low-taper root canals, as it is not possible to use the vertical condensation technique, which requires a greater taper [38].

From these analyses, it may be hypothesized that the PTNm technique created a more tapered preparation compared with TN, due to the geometric difference in the coronal portion of the instruments with taper 0.02 and 0.075 for TN and PTN systems, respectively. This is supported by the significant difference observed between groups for the RA parameter at the coronal level of analysis. This aspect seems in agreement with a recent study which reported that TN system touched a low percentage of root canal surface during shaping of lower molar mesial canals [9]. Moreover, the results of the RDR outcomes demonstrated that the techniques equally respected the original canal anatomy, without significant transportation, nor the risk of removing a considerable amount of dentin in correspondence of the furcation. Recently, Kabil et al. showed that TN- and PTN-shaping systems had similar transportation and centering abilities in the coronal and apical root canal portions [39]. However, in the present study, the analysis of the centroid shift suggests that PTNm was more centred at the apical level, although this was not significant. This may relate to the different techniques adopted for the shaping of the apical third, whereby the use of a shorter sequence and a manual NiTi instrument #25 could have resulted in a more conservative approach in the PTNm group. This aspect could be related to different outcomes in terms of post-operative pain and quality of life, but it was not investigated due to the ex vivo study limits [40,41]. 

In conclusion, the results of this study demonstrate that PTNm and TN exhibit comparable maintenance of the original canal anatomy, supporting the use of both shaping techniques for the instrumentation of curved lower molars mesial canals. 

## Figures and Tables

**Table 1:** Sample baseline characteristics of the 60 mesial canals were included in the study. ^a^ Apical diameters 1 mm from apical foramen. PTNm, modified ProTaper Next technique; TN, TruNatomy technique.

	PTNm (Mean ± SD)	TN (Mean ± SD)	*p*
Canal volume (mm^3^)	3.02 ± 0.59	3.01 ± 0.55	0.23
Canal surface area (mm^2^)	24.23 ± 1.94	24.59 ± 3.67	0.19
Apical diameter ^a^ (mm)	0.15 ± 0.09	0.18 ± 0.05	0.11

**Table 2:** 3D and 2D parameters utilized for post-shaping analysis in each group. Different superscript letters indicate statistical significance: ^a,b^ *p* < 0.05. For 2D parameters (centroid shift, RDR, and RA) significance was compared for the same level of analysis (coronal, middle, or apical). PTNm, modified ProTaper Next technique; RA, Ratio of Cross-Sectional Areas; RDR, Ratio of Diameters Ratios; TN, TruNatomy technique.

	Increase in Canal Volume (mm^3^)	Increase in Canal Surface Area (mm^2^)		Centroid Shift (mm^−1^)	RDR (Ratio)	RA (Ratio)
Group	Mean ± SD	Mean ± SD	Level of Analysis	Mean ± SD	Mean ± SD	Mean ± SD
			Coronal	1.25 ± 0.94 ^a^	0.60 ± 0.16 ^a^	1.82 ± 0.71 ^a^
**PTNm**	1.40 ± 0.80 ^a^	3.37 ± 2.17 ^a^	Middle	0.76 ± 0.47 ^a^	0.73 ± 0.18 ^a^	1.28 ± 0.22 ^a^
			Apical	0.83 ± 0.45 ^a^	0.76 ± 0.21 ^a^	1.33 ± 0.36 ^a^
			Coronal	0.77 ± 0.46 ^a^	0.61 ± 0.23 ^a^	1.30 ± 0.21 ^b^
**TN**	0.91 ± 0.44 ^a^	2.24 ± 1.48 ^a^	Middle	0.67 ± 0.29 ^a^	0.75 ± 0.14 ^a^	1.26 ± 0.16 ^a^
			Apical	1.45 ± 0.27 ^a^	0.68 ± 0.26 ^a^	1.29 ± 0.22 ^a^
